# Outcome and clinical changes in patients 3, 6, 12 months after a severe or major hand injury - can sense of coherence be an indicator for rehabilitation focus?

**DOI:** 10.1186/1471-2474-11-286

**Published:** 2010-12-22

**Authors:** Ragnhild I Cederlund, Eva Ramel, Hans-Eric Rosberg, Lars B Dahlin

**Affiliations:** 1 Division of Occupational Therapy and Gerontology, Department of Health Sciences, Lund University, Lund, Sweden; 2 Hand Surgery/Department of Clinical Sciences, Skåne University Hospital, Lund University, Malmö, Sweden

## Abstract

**Background:**

Our objective was to explore outcome and clinical changes in hand function, satisfaction in daily occupations, sleep disturbances, health and quality of life in consecutive patients after a severe or major hand injury. Our objective was also to investigate possible differences between groups according to severity of injury, presence of peripheral nerve injury and the patients' sense of coherence.

**Methods:**

A postal questionnaire, including demographic data, disabilities of the arm, shoulder and hand (DASH), QoL (SF-36), EuroQol (EQ-5D VAS), hand function (VAS), satisfaction in daily occupation (SDO), was sent out 3, 6 and 12 months after injury to 45 consecutive patients with a severe or major hand injury. Sense of coherence (SOC) was evaluated at 6 months. For the descriptive study, non-parametric tests were used since almost all results were measured with ordinal scales, the study sample was small, and most variables not normally distributed.

**Results:**

Almost all self-assessed aspects of hand function, satisfaction in daily occupations, health (DASH), and physical QoL (SF-36) improved statistically for the whole group over time. Large clinical improvement was seen for physical QoL and health, while a low or no improvement was observed for mental QoL, and cold sensitivity. Few differences were found between participants with a severe or major of hand injury or with or without a major nerve injury. No significant differences in demographic data were observed between participants with high or low SOC, but participants with low SOC showed significantly lower satisfaction in daily occupations, higher DASH scores, lower mental QoL, more sleep disturbances, and bodily pain. Correlation was found between SOC, and QoL, health and satisfaction in daily occupations.

**Conclusions:**

SOC had a significant influence on patients with a severe or major traumatic hand injury. Patients with lower SOC would probably benefit from extra support and help to master their daily life, indicating that sense of coherence is an indicator for future rehabilitation focus.

## Background

A continuous challenge in rehabilitation after a hand injury is to find evidence for the best treatment methods and routines to help patients back to their previous life and daily occupations. Patients with severe or major hand injuries need specialised medical and surgical treatment and continuous rehabilitation for a long period of time. The patients may experience reduced hand function, difficulties to perform daily occupations, and reduced health-related quality of life [1-4]. Performing daily occupations independently, and with satisfaction [5], may take time and include a variety of coping strategies [6,7]. Health-related quality of life has proven to be a valuable outcome to assess the impact of a disease or injury and mirrors the patients' own perspective [8]. Hand injuries may lead to substantial long-term disability [9-11] and psychological distress [6,12]. Not only the severity of an injury or disease can have a negative effect on outcome and a person's ability to master their daily occupations, but other factors, like the severity of injury and the patients' sense of coherence, may influence as well [13].

Sense of coherence (SOC) is a salutogenic theory introduced by Antonovsky [14,15]. It consists of three dimensions; comprehensibility, manageability and meaningfulness. With a salutogenic approach, factors influencing health are in focus in contrast to a pathogenic approach that focuses on the risk factors for disability. Antonovsky describes SOC as a disposition rather than a personality trait and claims that the way people view their life influences their health [15]. According to Eriksson and Lindström, SOC seems to be a health promoting resource [16] influencing QoL [17]. Higher SOC has also been reported to result in a more positive development of life satisfaction [18] in persons with mental health problems. In patients with moderate orthopedic injuries it has been shown that low SOC was associated with an increased risk of having less good clinical and functional outcome [19]. However, to our knowledge, there are no studies of SOC in relation to rehabilitation outcome in patients who have experienced a severe acute hand injury. SOC can be used as an indicator for selection of treatment, information given, level of individual motivation and psychological support [20]. We therefore regarded SOC as a possible factor of interest for future improvement in the rehabilitation of patients with serious hand injuries. Few studies have focused on the results and factors influencing outcome after the most serious hand injuries [21-23]. Our objective was to explore outcome and clinical changes in hand function, satisfaction in daily occupations, sleep disturbance, health and quality of life in consecutive patients after a severe or major hand injury. Our objective was also to investigate possible differences between groups according to severity of injury, presence of peripheral nerve injury and the patients' sense of coherence.

## Methods

The study design was quantitative using self-administrated questionnaires of consecutive participants who were included in an ongoing longitudinal study of patients with a severe or major hand injury being followed up after 3, 6, and 12 months.

### Participants

Participants able to communicate in Swedish and aged 16-65, with a major hand injury, based on Hand Injury Severity Score (HISS) with scores >100 [24,25] were recruited consecutively from our department during 2005-2007. In addition, due to a slow recruitment of participants with major hand injuries during the two years of inclusion, a number of randomly selected patients with severe injury (approximately every tenth patients) with HISS scores >50 were also included. The patients were given written and verbal information about the study and were asked to participate in the study. A written informed consent was obtained. Hand and forearm injuries caused by suicide attempt, and patients with a known severe psychiatric disorders or drug abuse were excluded. A total of 45 participants, 35 with a major and 10 with a severe hand injury were included in the study of which 36 were men and 9 were women. All patients were treated according to the latest evidence in the field of hand surgery and rehabilitation. The individual patients were treated according to the extent and nature of the injury and the decision of the consultant. The patients were followed up during three months or more. Rehabilitation included occupational- and physical therapy and contact with social worker. After 3 months all patients were allowed to use their hands with no restrictions. There was a single dropout: one patient's questionnaire was missed at 3 months. The characteristics of the participants are described (Table 1).

**Table 1:** Characteristics of the subjects included in whole group and subgroups.

Characteristics of the subjects	Whole group n = 45	Peripheral nerve injury n = 17	No peripheral nerve injury n = 28	High SOC ≥ 68 n = 23	Low SOC (< 68) n = 22
Age (y), median (range)	42 (16-64)	32 (16-58)	46 (19-64)	43 (19-64)	35 (16-63)
Gender, male/female (n)	36/9	13/4	23/5	17/6	19/3
HISS score, median (range)	154 (52-414)	140 (52-225)	159 (73-414)	140 (52-310)	161 (80-414)
Severity, major/severe injury (n)	35/10	11/6	24/4	16/7	19/3
Dominant/non-dominant hand injury (n)	21/24	8/9	13/15	9/14	12/10
Manual/non-manual worker (n)	31/14	14/3	17/11	14/9	17/5

### Assessment instruments

A self-administered and study specific questionnaire, including demographic data, perceived hand- and body functions and a number of standardised questionnaires was sent out to the participants by post at 3, 6, and 12 months post trauma. The questionnaire covered several areas of interest such as subjective pain, joint mobility, sensibility, grip strength, dexterity, sleep disturbance, cold sensitivity, satisfaction in daily occupations, health status, disability, and physical and mental quality of life. Questions concerning sense of coherence were included at 6 months.

#### Demographic data

Demographic data on age, gender, HISS score, severity of injury, dominant or non-dominant hand injury, and type of occupation were registered.

### Hand Injury Severity Score (HISS)

The Classification by Cambell and Kay (HISS) was used to classify the severity of the injuries. HISS is an objective anatomical assessment specifically designed for hand injuries. The hand injuries can be divided into four broad categories, such as "Minor" (least injury), "Moderate", "Severe", "Major" (worst injury). HISS is based on which tissues and which fingers that are affected by the injury [24,25].

#### Hand function, sleep disturbance and cold sensitivity

Six questions regarding hand and body function: such as pain, joint mobility, sensibility, grip strength, dexterity and sleep disturbance, were quantified using a visual analogue scale (VAS). The questions were formulated for example as "Describe your pain". The 10 cm scale extremes were 0 = no pain to 10 = worst possible pain or "Describe your grip strength, 0 = best possible grip strength to 10 = worst possible grip strength. Higher score indicates more reduced hand function. Cold sensitivity was also measured using the Cold Sensitivity Severity (CSS) scale [26,27], which includes four questions of situations in the home that may cause cold-related symptoms: "How much does cold bother your injured hand..." with a score ranging from 0 = not at all to 10 = extreme. Total score ranging from 0-40.

#### Daily occupations

Satisfaction in daily occupations was measured with the *Satisfaction with Daily Occupations (SDO) *instrument, a screening tool with demonstrated good psychometric properties [5,28]. The instrument was originally developed for people with mental disorders, but has satisfactorily been used for patients with scleroderma [29]. SDO consists of nine questions covering work (4 items), leisure activities (2 items), domestic tasks (2 items), and self-care (1 item). Each question consists of a two-part answer. One question, participation in community-based activity centres, was not used in this study as it was not relevant to this group of patients. Two types of score can be calculated with SDO. Only one score, satisfaction on daily occupations, was used. The scores ranges from 1 = worst possible satisfaction to 7 = best possible satisfaction. The scores are expressed as a sum of satisfaction ratings. Higher score indicates higher satisfaction for the person and the possible range (based on 8 questions) is 8-56.

#### Health and quality of life

To measure health outcome, the EuroQol EQ-5D was used [30,31]. The EQ-5D is divided into three parts; descriptive system, visual analogue scale (VAS), and index. The EQ-5D VAS measuring perceived health was used. The VAS scale is a 20 cm vertical thermometer and has an endpoint of 100 (best imaginable health state) at the top and 0 (worst imaginable health state) at the bottom. The patient rates his or her current health by drawing a line from the box marked "your own state of health today" to a chosen point on the EQ-5D VAS thermometer. Higher score indicates a better health.

The *disabilities of the arm, shoulder and hand (DASH) *is a region-specific and standardized questionnaire [32,33]. It is based on the WHO model of health and assess impairments, and activity limitations and participation restrictions for both work and leisure activities. The questionnaire covers 21 daily activities, and 9 symptom questions and questions related to self-image and social functioning. Response options range from 0: no difficulty to 5: unable. The DASH score ranges from 0-100 with 0 indicating no disability and 100 signifying most severe disability.

The Medical Outcome Study (MOS) 36-item short form health survey (SF-36) is a self-administered generic instrument that measures health-related quality of life [34]. There are eight domains divided into a physical component scale (physical functioning, role-physical, bodily pain, general health) and a mental component scale (vitality, social functioning, role-emotional, mental health). The results within each domain ranges from 0 (poor health) to 100 (optimal health) and can be reported separately, or summed as physical- and mental domain score and compared with normative data. Higher score indicates a better health status. The Swedish version was used in this study [8]. The patients were compared to a population norms (Sweden) from age and gender matched individuals (n = 1180) obtained from the Swedish SF-36 database for population norms (n = 8930) [8].

#### Sense of coherence

Sense of coherence (SOC) was assessed with Antonovsky's short 13-item scale [15] in a Swedish version [35]. The SOC questionnaire reflects a person's capacity to cope in a stressful situation and a disposition towards seeing the world as comprehensible, manageable and meaningful [15]. The scores on each item ranges from 1 (never) to 7 (very often). The total score range is 13-91. High scores indicate a strong SOC. Normative data from published studies using SOC-13 ranges from mean values (MD) 58.5 (12.1) to 68.7 (10.0) [36]. The median SOC value of the whole group in this study was 68. A pre-injury measure of SOC was not relevant in the present study as the patients were referred acutely. Therefore, we chose to assess SOC at six months due to no restrictions in using their hands and that half of the patients were back at work.

### Data analysis

Almost all variables are presented as median (range). However, to present descriptive outcome for SF-36 mean values (standard deviation, SD) were used to facilitate comparisons with previous studies. For analyses non-parametric tests were used since almost all items were measured with ordinal scales, the study sample was small, and most variables not normally distributed. Friedman's test was used to detect differences over time (3-6-12 months) with the VAS-score, CSS, SDO, EQ-5D VAS, DASH and SF-36. Mann-Whitney U-test was used to detect differences between subgroups. Wilcoxon signed rank test was used to detect differences between patients with high/low SOC and the population norms. Z-scores (individual value minus mean value of populations norms divided by SD from the population norms) were calculated for all SF-36 parameters at 12 months. Responsiveness of hand function, sleep disturbance, satisfaction in daily occupations and health and quality of life was expressed as effect size. Effect size for SF-36 was calculated with mean change in score, between 3-12 months divided by standard deviation of the three months score [37]. As an alternative, effect size was also calculated as median change in score divided by interquartile range. According to Cohen's criterion (p. 40), an effect size of ≤ 0.20 is considered small, 0.50 moderate, and 0.80 large clinical changes [38]. For correlations, Spearman (r_s_) values were calculated. A correlation above 0.75 is generally described as good to excellent relationship, 0.50-0.75 moderate to good, 0.25 to 0.5 fair and 0.00-0.25 little or no relationship [39]. A multiple regression analysis was done to evaluate factors explaining change in different variables at 6 and 12 months. The median SOC score of 68 was used to dichotomize the study population in order to reveal any differences between high/low SOC. The statistical analysis was performed with SPSS version 17.0 for Windows (SPSS Inc., Chicago, IL, USA). Eighteen measured areas of interest were analysed at three different occasions; 3, 6 and 12 months after the injury and 54 statistical tests were performed. The significance level was set at p ≤ 0.05. Considering a risk of mass significance the results at a stricter significance level of p ≤ 0.01 is pointed out.

### Ethics

Approval of the study was obtained from the Ethics Committee, Lund University, Sweden (714/2004).

## Results

### Outcome and clinical changes 3, 6 and 12 months after a hand injury

#### The whole group

Self assessed outcome, such as joint mobility, sensibility, grip strength, and dexterity, improved significantly from 3 to 12 months for the whole group (Table 2). Satisfaction with daily occupations (SDO), health (EQ-5D VAS, DASH), and quality of life (SF-36), especially physical functioning, role physical and bodily pain, also improved significantly (Tables 2 and 3). No significant differences were noticed for pain, sleep disturbances, cold sensitivity, general health or any of the four mental components in SF-36.

**Table 2:** Subjective outcome concerning hand function, satisfaction in daily occupations, and health (n = 45).

	Major and severe hand injuries n = 45 (median, range)				
	Three months n = 44	Six months n = 45	Twelve months n = 45	**Group differences**^**a**^ (3-12 months)	**Effect size**^**b**^ 3 m vs 12 m
Pain (VAS)	2.7 (0-9.5)	2.4 (0-8.2)	2.0 (0-8.4)	0.270	0.2
Joint mobility (VAS)	6.2 (1.5-10)	5.4 (0-10)	4.6 (0-10)	0.019	0.5
Sensibility (VAS)	6.5 (0.5-10)	5.2 (0.7-10)	4.3 (0-10)	0.004	0.7
Grip strength (VAS)	7.2 (0.6-10)	6.1 (0.5-10)	5.4 (0.2-10)	0.002	0.6
Dexterity (VAS)	8.0 (1-10)	6.5 (0-10)	5.8 (0-10)	0.005	0.7
Sleep disturbance (VAS)	1.0 (0-10)	0.5 (0-10)	0.4 (0-8.8)	0.402	0.1
Cold sensitivity (CSS)	15 (0-33)	15 (0-31)	13 (0-40)	0.686	0.0
Satisfaction in daily occupations (SDO)	35 (12-50)	38 (16-56)	42 (12-56)	0.001	0.4
Health status (EQ-05 VAS)	70 (1-100)	70 (15-100)	79 (14-100)	< 0.001	0.3
Disabilities in the arm, shoulder, hand (DASH)	30 (1.7-79.2)	18 (0.8-60.8)	13 (0-56.7)	< 0.001	0.9

^a ^Friedman's test. ^b^Effect size measure for VAS, CSS, SDO, EQ-05VAS and DASH was calculated with median change in score divided by interquartile range. VAS (0-10) higher score indicates more reduced hand function, CSS (0-40) higher score indicates more reduced hand function, SDO (8-56) higher score indicates higher satisfaction, EQ-05VAS (0-100) higher score indicates a better health and DASH (0-100) higher score indicates more severe disability.

**Table 3:** Subjective outcome concerning quality of life (n = 45).

Quality of life (SF-36)	Three months n = 44	Six months n = 45	Twelve months n = 45	**Group Differences**^**a**^ P-value	**Effect size**^**b**^ 3 m vs 12 m	**Population norms**^**c**^ SF-36	**Z score**^**d **^**for comparison 12 m vs population norms**
					
	Mean score (SD)						
Physical Functioning	76 (13.9)	84 (12.4)	87 (12.6)	< 0.001	0.8	91 (17.3)	-0.2 (0.73)
Role Physical	22 (31.9)	47 (41.0)	60 (40.0)	< 0.001	1.2	87 (28.4)	-1.0 (1.46)
Bodily Pain	60 (23.3)	70 (20.0)	72 (23.8)	0.006	0.5	77 (25.4)	-0.2 (0.95)
General Health	77 (17.1)	76 (20.8)	77 (22.0)	0.978	0.0	78 (21.0)	-0.0 (1.01)
Vitality	59 (19.5)	63 (22.2)	64 (20.0)	0.444	0.2	71 (22.0)	-0.3 (0.92)
Social Functioning	84 (20.7)	84 (22.4)	89 (19.2)	0.120	0.2	90 (18.6)	-0.1 (1.02)
Role Emotional	54 (44.3)	67 (42.0)	71 (38.0)	0.127	0.4	89 (25.6)	-0.7 (1.49)
Mental Health	72 (20.4)	72 (23.3)	75 (20.7)	0.592	0.1	82 (18.1)	-0.4 (1.16)

^a^Friedman's test. ^b^Effect size for SF-36 was calculated with mean change in score, three months to follow up after twelve months, divided by standard deviation of the three months score. ^c^Population norms were obtained from the Swedish SF-36 database for population norms (n = 8930) and were age and gender matched with individuals (n = 1180). ^d^Z-scores (individual value minus mean value of populations norms divided by SD from the population norms) were calculated for all SF-36 parameters at 12 months. SF-36 (0-100) higher score indicates a better health status 0 (poor health) to 100 (optimal health).

Largest clinical changes (effect size ≥ 0.8) for the whole group were found in role physical and physical functioning in SF-36 (Table 3), and in DASH (Table 2). Moderate changes (≥ 0.5) were observed in joint mobility (VAS), sensibility, grip strength, dexterity and bodily pain (SF-36) (Table 2). The patients' outcome from SF-36 at 12 months were compared to a normal population randomly selected, gender and age matched reference group (n = 1180) from the Swedish SF-36 database for population norms (n = 8930). Despite a large positive clinical change (Effect size 1.2) in role physical for the whole group, it was that domain that differed most from population norms (Z-score -1.0) (Table 3). Another domain that differed from population norms (Z-score -0.7) was role emotional.

#### Subgroups

The whole group (n = 45) was divided into subgroups with respect to sense of coherence, presence of a major peripheral nerve injury (median, ulnar or radial nerve) and severity of injury (HISS).

##### Sense of coherence

Between the subgroups with high or low SOC, significant differences in outcome were found in 15 of the 18 measured areas of interest (32/54 variables) at 3, 6, 12 months follow-up. Participants with low SOC had on the whole a worse outcome compared to patients with a high SOC in all variables (Tables 4 and 5). Sensibility, dexterity, and sleep disturbances (VAS), but also satisfaction in daily occupations (SDO), health status (EQ-5D VAS) and most variables in mental quality of life (SF-36), showed better values in patients with high SOC. No differences were found in cold sensitivity. Participants with a high sense of coherence (SOC) showed large clinical changes (0.8) at 12 months compared to baseline in role physical and satisfaction in daily occupations. Large clinical changes were in role physical and bodily pain among participants with low SOC (Table 5). Role physical and role emotional concerns perceived difficulties performing work and other regular daily activities due to physical or mental problems [8]. The patients with high and low SOC differed with respect to the majority of the domains in SF-36 (Table 5). We found differences in 5/8 domains in SF-36 in participants with low SOC compared to the population norms, while only 1/8 domains among the patients with high SOC differed (Table 5). When the significant level was set at p ≤ 0.01, 12 of the 18 measured areas of interest (23/54 variables) were still significant at 3, 6, 12 months follow-up when compared to high/low SOC (Tables 4 and 5).

**Table 4:** Differences between patients with high (≥68) or low (< 68) SOC and subjective outcome concerning hand function, satisfaction in daily occupations, and health.

	Median score (range)		**Group Differences**^**a**^ P-value	**Effect size**^**b**^ 3 m vs 12 m	
	
	High SOC (n = 23)	Low SOC (n = 22)		High SOC	Low SOC
Pain (VAS)					
3 months	1.9 (0-9.5)	4.0 (0-7.9)	0.059	-	-
6 months	1.7 (0-5.3)	3.0 (0-8.2)	0.027	-	-
12 months	1.8 (0-5.0)	2.3 (0-8.4)	0.294	0.0	0.5
Joint mobility (VAS)					
3 months	5.9 (2-9.5)	6.5 (1.5-10)	0.383	-	-
6 months	5.2 (0-9.5)	5.5 (1.2-10)	0.768	-	-
12 months	4.5 (0.5-8.8)	5.3 (0-10)	0.650	0.4	0.3
Sensibility (VAS)					
3 months	5.1 (2.2-10)	7.4 (0.5-9.8)	0.058	-	-
6 months	4.0 (0.7-8.5)	6.3 (1-10)	0.007	-	-
12 months	4.0 (0-9.5)	5.7 (0-10)	0.388	0.3	0.6
Grip strength (VAS)					
3 months	5.4 (0.6-9.5)	7.5 (2-10)	0.043	-	-
6 months	6.0 (1.5-10.0)	6.2 (0.5-10)	0.742	-	-
12 months	5.3 (1.6-9.0)	5.5 (0.2-10)	0.750	0.0	0.6
Dexterity (VAS)					
3 months	6.6 (1-9.5)	8.2 (3.1-10)	0.012	-	-
6 months	6.4 (0-10)	7.2 (3-10)	0.433	-	-
12 months	5.2 (0-9.0)	6.9 (4-10)	0.007	0.3	0.8
Sleep disturbance (VAS)					
3 months	0.5 (0-7.2)	3.0 (0-10)	0.028	-	-
6 months	0.2 (0-8.4)	2.4 (0-10)	0.015	-	-
12 months	0.0 (0-7.8)	1.7 (0-8.8)	0.003	0.1	0.2
Cold sensitivity (CSS)					
3 months	14 (0.8-32.5)	16 (0-33.2)	0.851		
6 months	15 (0-26.2)	16 (0-31)	0.829		
12 months	13 (0-40)	14 (1.2-29.8)	0.547	0.1	0.1
Satisfaction daily occupations (SDO)					
3 months	38 (12-50)	32 (12-44)	0.007	-	-
6 months	43 (23-56)	35 (16-52)	0.014	-	-
12 months	45 (29-56)	40 (12-56)	0.030	0.6	0.5
Health status (EQ-05 VAS)					
3 months	79 (30-100)	60 (0-81)	0.022	-	-
6 months	80 (50-100)	67 (15-85)	0.002	-	-
12 months	90 (49-100)	72 (14-100)	0.002	0.4	0.5
Disabilities in the arm, shoulder, hand (DASH)					
3 months	30 (1.7-52)	31 (9.2-79.2)	0.265	-	-
6 months	12 (0.8-36.7)	22 (7.5-60.8)	0.020	-	-
12 months	8.3 (0-45)	16 (1.7-56.7)	0.069	0.9	0.5

^a ^Friedman's test. ^b ^Effect size measure for VAS, CSS, SDO, EQ-05VAS and DASH was calculated with median change in score divided by interquartile range. VAS (0-10) higher score indicates more reduced hand function, CSS (0-40) higher score indicates more reduced hand function, SDO (8-56) higher score indicates higher satisfaction, EQ-05VAS (0-100) higher score indicates a better health and DASH (0-100) higher score indicates more severe disability.

**Table 5:** Differences between patients with high (≥68) or low (< 68) SOC concerning quality of life.

Quality of life (SF-36)	(Population norm ^a ^[SD]) Mean score (SD)			**Effect size**^**b**^ 3 months vs 12 months		Z-score ^c ^for comparison with population norms Mean (SD)		
	
	High SOC (n = 23)	Low SOC(n = 22)	Group differences	High SOC	Low SOC	High SOC	Low SOC	P-values ^d^
Physical functioning	(91 [17.3])							
3 months	76 (14.7)	76 (13.4)	0.922	-	**-**	**-**	**-**	
6 months	87 (11.6)	81 (12.7)	0.064	-	**-**	**-**	**-**	
12 months	90 (8.65)	84 (15.1)	0.117	1.0	0.5	-0.0 (0.50)	-0.5 (0.87)*	0.061
Role Physical	(87 [17.3])							
3 months	30 (35.9)	14 (25.7)	0.157	-	-	**-**	**-**	
6 months	57 (40.1)	38 (40.6)	0.122	-	-	-	-	
12 months	77 (37.6)	42 (34.8)	0.001	1.3	1.1	-0.4 (1.39)	-1.6 (1.27)*	0.002
Bodily pain	(77 [25.4])							
3 months	72 (22.0)	48 (18.2)	0.001	-	-	-	-	
6 months	80 (19.0)	59 (24.5)	0.005	-	-	-	-	
12 months	80 (19.6)	64 (25.5)	0.028	0.4	0.9	0.1 (0.76)	-0.5 (1.03)	0.035
General Health	(78 [21.0])							
3 months	83 (14.1)	70 (17.8)	0.014	-	-	-	-	
6 months	83 (17.5)	69 (22.1)	0.036	-	-	-	-	
12 months	84 (17.8)	69 (24.0)	0.042	0.0	0.0	0.3 (0.81)	-0.3 (1.12)	0.094
Vitality	(71 [22.0])							
3 months	67 (19.1)	51 (16.7)	0.007	-	-	-	-	
6 months	73 (19.1)	52 (20.4)	0.002	-	-	-	-	
12 months	74 (19.6)	53 (14.4)	< 0.001	0.4	0.1	0.3 (0.81)	-0.8 (0.66)*	< 0.001
Social Functioning	(90 [18.6])							
3 months	91 (14.1)	76 (23.7)	0.011	-	-	-	-	
6 months	94 (15.0)	74 (24.8)	0.003	-	-	-	-	
12 months	98 (7.71)	80 (23.7)	0.008	0.4	0.2	0.3 (0.44)*	-0.5 (1.26)	0.021
Role Emotional	(89 [25.6])							
3 months	62 (44.0)	46 (44.1)	0.251	-	-	-	-	
6 months	75 (37.9)	58 (45.1)	0.148	-	-	-	-	
12 months	83 (36.1)	59 (37.0)	0.017	0.5	0.3	-0.3 (1.42)	-1.2 (1.44)*	0.035
Mental Health	(82 [18.1])							
3 months	82 (14.9)	61 (20.2)	< 0.001	-	-	-	-	
6 months	83 (16.7)	61 (24.3)	0.001	-	-	-	-	
12 months	85 (14.4)	64 (21.0)	0.001	0.2	0.1	0.1 (0.81)	-1.0 (1.19)*	0.001

^a ^Population norms were obtained from the Swedish SF-36 database for population norms (n = 8930) and n = 45 were age and gender matched with individuals (n = 1180). ^b ^Effect size for SF-36 was calculated with mean change in score, three months to follow up after twelve months, divided by standard deviation of the three months score. ^c ^Z-scores (individual value minus mean value of populations norms divided by SD from the population norms) were calculated for all SF-36 parameters at 12 months. SF-36 (0-100) higher score indicates a better health status 0 (poor health) to 100 (optimal health). ^d ^Differences between high and low SOC (Mann Whitney). * Different from the population norms (Wilcoxons Signed Ranks Test).

##### Presence of a major peripheral nerve injury

The participants were divided into groups with (n = 17) or without (n = 28) a major peripheral nerve injury in the arm (median, ulnar and radial nerve). Between the subgroups significant differences in outcome were found in four of the 18 measured areas of interest (4/54 variables) at 3, 6, 12 months follow-up. Participants with no peripheral nerve injury had a better recovery in sensibility (VAS p-value at 3 months = 0.02), joint mobility (VAS p-value at 12 months = 0.01), grip strength (VAS p-value at 12 months = 0.04) and social functioning (SF-36 p-value at v12 months = 0.02). When the significant level was set at p ≤ 0.01, one of the 18 measured areas of interest (1/54 variables) was still significant when compared to peripheral nerve injury/no nerve injury.

##### Severity of hand injury

The participants were divided into two groups with severe (HISS > 50) and major (HISS > 100) hand injury. Between the subgroups with severe or major hand injury significant differences in outcome were found in six of the 18 measured areas of interest (10/54 variables) at 3, 6, 12 months follow-up. Participants with a severe hand injury had a better recovery in health status (EQ-5D VAS p = 0.05 at 3, 6 and 12 months; DASH p = 0.01 at 3 and 6 months), physical functioning (SF-36 p = 0.02 at 6 and 12 months), role physical (SF-36 p = 0.03 at 12 months), mental health (SF-36 p = 0.005 at 3 months) and grip strength (VAS p = 0.05 at 12 months) than patients with a major injury. When the significant level was set at p ≤ 0.01 two of the 18 measured areas of interest (3/54 variables) were still significant when compared to severe/major hand injury.

#### Correlations with SOC

Statistically significant correlation in the whole group between SOC and mental component scale (MCS p = 0.001; r_s _0.53), EuroQol (EQ-5D VAS (p = 0.001; r_s _0.53), satisfaction in daily occupations (SDO p = 0.001; r_s _0.49), and physical component scale (PCS p = 0.003; r_s _0.43) were observed. No other significant correlations were found. Correlations were also performed among the variables with significant differences between high and low SOC (Tables 4 and 5). At 6 months 13/18 variables showed significant correlations ranging from -0.30 to 0.67. The corresponding values at 12 months were -0.35 to 0.64. An additional multiple regression analyses (results not shown) showed that SOC was the main factor explaining the outcome at 6 and 12 months, although age also influenced health (EuroQol), sleep disturbances (VAS) and physical functioning (SF-36).

## Discussion

One year after repair and reconstruction of a severe or major hand injury almost all self-assessed physical aspects of hand function, satisfaction in daily occupations, health and physical quality of life had improved. HISS was used to identify patients with a major or severe hand injury and is commonly used to assess the severity of a hand injury and to reflect long term outcomes [21-23]. A low or no improvement was observed for mental QoL, and cold sensitivity. Noticeably, few differences were found between participants with a severe or major hand injury or between participants with or without a major peripheral nerve injury. An injury to a major nerve trunk in the forearm and a complex hand injury seem to create fairly equal problems for hand function, the patient's daily occupation and quality of life. The results showed significant correlations between sense of coherence, quality of life, health, and satisfaction in daily occupations. Our results confirm the results from other studies showing correlations between mental health, and daily occupations [40-42]. However, the most surprising findings were the influence that sense of coherence had on the 54 self-assessed variables in the questionnaire.

There were numerous differences in outcome between patients with a high or low SOC. Patients with a high SOC generally assessed their situation in a more favorable way than patients with a low SOC score. In related areas, in patients with orthopaedic conditions with low SOC, signs of depression and less control over life were associated with a risk of having less positive clinical and functional outcome one year after the injury [19]. In primary health care, a low SOC had a significant effect on frequent attendance (a disproportional use of health care), possibly due to inadequate coping strategies [13]. Antonovsky [15] claimed that a person's SOC is stable when having reached adult age, which was why we only assessed this once and at 6 months when the acute phase was over but the patient still wasn't fully recovered in most cases. More recent research [18,43] now suggest that it is possible to have different SOC scores according to different situation in life and that SOC may improve with intervention. It would therefore be of interest to use SOC in rehabilitation after hand injuries. Our findings seem to suggest that SOC in addition to patient's mental health needs to be considered further when planning rehabilitation of patients with serious hand trauma. Many different coping strategies and flexibility in using them seem to be important for a good and fast recovery [6,44]. When SOC is known this can be yet another way to understand the patients' coping ability. It can point out the need for extra patient support in order to assist in the rehabilitation process.

A prominent feature was that physical functioning (SF-36) improved from three to twelve months in the whole group up to the level of population norms. Furthermore, dexterity, sensibility, grip strength, and joint mobility all improved during the follow up. The aim of this study was not to examine the whole range of therapy that was performed with these patients, but knowing that mostly evidence based treatments were used in rehabilitation; thus, the present findings are not surprising. We found very few differences between patients with or without major nerve injury.

An important finding was that mental quality of life did not improve during the twelve month follow up and it did not reach the population norms. This shows the impact a severe or major hand injury has on the individual in contrast to a less severe injury, like a carpal tunnel syndrome [45]. In our study these severely injured patient's mental needs were not addressed enough. Emotional problems should be considered more in rehabilitation and questionnaires focusing on post traumatic stress syndrome and depression [46,47] included in the standard and routine assessments.

Cold sensitivity did not improve over the 12 month period for the whole group. No difference was seen between patients with a high or low SOC or within the other subgroups. The short Cold Sensitivity Severity (CSS) scale to assess cold sensitivity was used. The test is not season specific and reflects a range of severity of exposure in the home. CSS was used as our focus was to get an overall perception of the phenomenon. If the purpose is to define abnormal sensitivity and find out details about recovery and strategies relieving symptoms, then a more detailed questionnaire, such as the Cold Intolerance Symptom Severity score (CISS), may be recommended [48]. Our results are not surprising in view of the fact that cold sensitivity does not change over a long follow up time (up to a mean of 73-120 months) after a peripheral nerve injury [49]. A routine single question on cold sensitivity should always be asked for screening of severity. Behavioural treatment of cold sensitivity can relieve symptoms [50] but most important is to offer patient information on reliving strategies to help adaptation for daily life [51].

To understand the consequences on daily occupations after a serious hand injury, two instruments, the SDO and the DASH, were used. The DASH score correlates with the hand injury severity score (HISS) [21-23]. Outcome, based on these instruments, improved during follow up, but the results differed statistically at 12 months for SDO between the groups with high or low SOC. Improvements during follow up in daily occupations after a hand or forearm injury are in accordance with other studies [52,53]. The DASH score improved significantly over time (from median value 31 to 13), and an improvement of DASH with more than 10 points is considered clinically relevant [54]. Our results indicate that patients experienced less disability one year after the injury, but patients with low SOC experienced less satisfaction in daily occupations. Daily occupations is a complex construct and some instruments focus on doing a daily occupation (ability/disability), others on how the doing is perceived (satisfaction in daily occupation) and some concern both [28]. There is an important distinction between ability and satisfaction. Our recommendation is to include an assessment instrument focusing on satisfaction in daily occupations to assist the occupational therapist how to support the patients in a health promoting daily life. The SDO or Canadian occupational performance measure (COPM) [55] could be included in the standard and routine assessments.

Pain, as assessed by VAS, did not improve over the follow up. However, bodily pain improved, indicating that the pain may be located to the hand. Grip strength did also improve over the 12 month follow up with a slight difference between the two groups of severe or major hand injuries. Participants with a low SOC scored less grip strength on VAS at 3 months. In addition, dexterity differed at 3 and 12 months. A future rehabilitation goal is how to improve grip strength and dexterity for these patients. Therapeutic activities mimicking daily activities have been found to improve the hand more effectively [56]. We may consider focusing more on grip strength and dexterity after 3 months when tissues have healed and make specific suggestions how to improve the hand grip while using the hands in meaningful daily occupations.

We found no change in sleep disturbance over the 12 months period. On the other hand the median value for sleep disturbance was relatively low. However, the sleep disturbance was more pronounced in participants with a low SOC. This makes sense as persons with low SOC have more difficulties in coping with their injury. They may worry more, give up earlier and use ineffective problem-solving strategies [15]. In addition, the SF-36 revealed worse scores in all variables in participants with a low SOC. This might reflect other health problems than the hand injury [13] which may indicate the need for a more general rehabilitation strategy and not only concentrate on specific rehabilitation of the hand.

### Study limitations

The study sample was small, although we included patients for two years. Also the severity and the rather unique character of each individual's injury make it difficult to generalize to other groups of patients, and some of the results especially around SOC need to be studied further. SOC was for obvious reasons first measured after the injury had occurred and only measured once (after six months). When planning the study we argued that the SOC score after such a long time was both true and also stable according to Antonovsky's views [15]. Later research indicates that SOC may change and thus increase with age [43] and can improve with intervention [18]. If SOC changes because of a sudden injury, which is unclear, this needs to be studied in patients with a hand injury where a baseline SOC is available and can be followed up over the time of recovery. In addition, one has to consider that these patients, all with severe or major injuries according to HISS, received individualized treatment of their specific injuries, which may also influence the outcome. For rehabilitation reasons it is of outmost interest that SOC in longitudinal studies has shown a comparatively high predictability [43], although there is still a problem with interpreting the individual position on the SOC scale on the healthy/unhealthy continuum. In this study the median value of SOC was used as a cut point for later analysis of the whole group, but this value should not be considered a generalized recommended cut point for screening individuals. It can however add to the already existing knowledge of mean values in different patient groups and patients with "lower" scores can be identified for more intense support during the rehabilitation process. We chose a significance level of p ≤ 0.05, but if a stricter significance level p ≤ 0.01 is chosen there were still significant differences between high/low SOC subgroups in outcome.

## Conclusion

Most physical aspects of hand function, satisfaction in daily occupations, and health improved a year after repair and reconstruction among all participants with a severe or major hand injury, while mental health improved marginally and cold sensitivity did not improve at all. The perceived physical health-related quality of life (SF-36) approached the population norms in Sweden, particularly for participants with a high SOC. Participants with low SOC showed significantly lower satisfaction in daily occupations, higher disability (DASH), lower mental QoL, more sleep disturbances, and bodily pain. We conclude that SOC had a significant influence on participants with a severe or major hand injury and our results indicate that sense of coherence is an indicator for future rehabilitation focus. Patients with lower SOC may have inadequate coping strategies and need extra support and help to master their daily life after a serious hand injury. These patients may need more meticulous and guided rehabilitation, such as developing and using more coping strategies in their daily activities. They may benefit from more frequent visits and follow-ups, by using both verbal and written patient-information and education, participation in rehabilitation groups to meet other patients in the same situation and also meeting expert personnel focusing on their mental health.

## Competing interests

The authors declare that they have no competing interests.

## Authors' contributions

RC participated in the design of the study, collected the data, performed the statistical analyses, and drafted the manuscript. ER participated in data preparations and revising the manuscript. HER prepared all HISS score from patient journals and revising the manuscript. LD participated in the design of the study and in the progress and revision of the manuscript. All authors read and approved the final manuscript.

## Pre-publication history

The pre-publication history for this paper can be accessed here:

http://www.biomedcentral.com/1471-2474/11/286/prepub

## References

[B1] Bialocerkowski AE Difficulties associated with wrist disorders--a qualitative study Clinical Rehabilitation 2002 16 4 429 440 10.1191/0269215502cr516oa 12061478

[B2] Graham B Schofield M Self-reported symptoms of cold intolerance in workers with injuries of the hand Hand (N Y) 2008 3 3 203 209 18780096 10.1007/s11552-008-9116-0 PMC2525881

[B3] Gustafsson M Ahlstrom G Problems experienced during the first year of an acute traumatic hand injury - a prospective study Journal of Clinical Nursing 2004 13 8 986 995 10.1111/j.1365-2702.2004.01019.x 15533105

[B4] Poerbodipoero S Steultjens M van der Beek A Dekker J Pain, disability in daily activities and work participation in patients with traumatic hand injury The British Journal of Hand Therapy 2007 12 2 40 47

[B5] Eklund M Satisfaction with daily occupations: a tool for client evaluation in mental health care Scand J Occup Ther 2004 11 3 136 142 10.1080/11038120410020700

[B6] Cederlund R Thorén Jonsson A-L Dahlin LB Coping strategies in daily occupations 3 months after a severe or major hand injury Occupational Therapy International 2010 17 1 1 9 10.1002/oti.287 20101624

[B7] Gustafsson M Persson L-O Amilon A A qualitative study of coping in the early stage of acute traumatic hand injury Journal of Clinical Nursing 2002 11 594 602 10.1046/j.1365-2702.2002.00657.x 12201886

[B8] Sullivan M Karlsson J Ware J The Swedish SF-36 health survey: evaluation of data quality, scaling assumptions, reliability and construct validity across general populations in Sweden Social Science Medicine 1995 41 1349 1358 10.1016/0277-9536(95)00125-Q 8560302

[B9] Bruyns C Jaquet J-B Schreuders T Kalmijn S Kuypers P Hovius S Predictors for return to work in patients with median and ulnar nerve injuries Journal of Hand Surgery (American Volume) 2003 28A 28 34 10.1053/jhsu.2003.50026 12563634

[B10] Novak C Anastakis D Beaton D Katz J Patient-reported outcome after peripheral nerve injury Journal of Hand Surgery (American Volume) 2009 34A 281 287 10.1016/j.jhsa.2008.11.017 19181228

[B11] Rosberg H Carlsson K Dahlin L Prospective study of patients with injuries to the hand and forearm: costs, function, and general health Scandinavian Journal of Plastic Reconstructive Surgery and Hand Surgery 2005 39 6 360 369 10.1080/02844310500340046 16298809

[B12] Jaquet J-B van der Jagt I Kuypers P Schreuders T Kalmijn S Hovius S Spagetti wrist trauma: functional recovery, return to work, and psychological effects Journal of Plastic and Recontructive Surgery 2005 115 6 1609 1617 10.1097/01.PRS.0000160697.41738.EA 15861065

[B13] Bergh H Baigi A Fridlund B Marklund B Life events, social support and sense of coherence among frequent attenders in primary health care Public Health 2006 120 229 236 10.1016/j.puhe.2005.08.020 16337979

[B14] Antonovsky A Health, stress and coping 1979 San Francisco: Jossey-Bass

[B15] Antonovsky A Unraveling the mystery of health. How people manage stress and stay well 1987 San Francisco: Jossey-Bass

[B16] Eriksson M Lindström B Antonovsky's sense of coherence scale and the relation with health: a systematic review Journal of Epidemiology and Community Health 2006 60 376 381 10.1136/jech.2005.041616 16614325 PMC2563977

[B17] Eriksson M Lindström B Antonovsky's sense of coherence scale and it's relation with quality of life: a systematic review Journal of Epidemiology and Community Health 2007 61 938 944 10.1136/jech.2006.056028 17933950 PMC2465600

[B18] Langeland E Wahl A Kristoffersen K Nortvedt M Hanestad B Sense of coherence predicts change in life satisfaction among home-living residents in the community with mental health problems: a 1-year follow-up study Quality of life reserach 2007 16 939 946 10.1007/s11136-007-9199-z 17404897

[B19] Ristner G Andersson R Johansson L Johansson S-E Ponzer S Sense of coherence and lack of control in relation to outcome after othopaedic injuries Injury: International Journal of the Care of the Injured 2000 31 751 756 10.1016/s0020-1383(00)00115-7 11154742

[B20] Gassne J Salutogenes, Kasam och socionomer 2008 Lund: Lund university

[B21] Bueno RJ Neumeister M Outcomes after mutilating hand injuries: review of the litterature and recommendations for assessment Hand Clinics 2003 19 1 193 204 10.1016/S0749-0712(02)00142-7 12683456

[B22] Eriksson M Karlsson J Steen Karlsson K Dahlin LB Rosberg HE Economic consequences of accidents to hands and forearms by logsplitters and circular saws Journal of plastic and Recontructive Surgery and Hand Surgery 2010 in press 10.3109/2000656X.2010.542654 21446797

[B23] Saxena P Cutler L Feldberg L Assessment of the severity of hand injuries using "hand injury severity score", and its correlation with the functional outcome Injury: International Journal of the Care of the Injured 2004 35 511 516 10.1016/S0020-1383(03)00211-0 15081330

[B24] Campbell DA Kay SP The Hand Injury Severity Scoring System Journal of Hand Surgery (British and European Volume) 1996 21 3 295 298 10.1016/S0266-7681(05)80187-1 8771461

[B25] Urso-Baiarda F Lyons RA Laing JH Brophy S Wareham K Camp D A prospective evaluation of the modified hand injury severity score in predicting return to work International Journal of Surgery 2008 6 45 50 10.1016/j.ijsu.2007.09.001 18029238

[B26] Carlsson I Cederlund RHP Lundborg G Rosén B Hand injuries and cold sensitivity. Reliability and validity of cold sensitivity questionnaires Disability and Rehabilitation 2008 30 25 1920 1928 10.1080/09638280701679705 19061118

[B27] McCabe S Mizgala C Glickman L The measurement of cold sensitivity of the hand Journal of Hand Surgery American Volume 1991 16 1037 1040 10.1016/S0363-5023(10)80065-6 1748748

[B28] Eklund M Gunnarsson B Content validity, clinical utility, sensitivity to change and discriminant ability of the Swedish satisfaction with daily occupations (SDO) instrument: a screening tool for people with mental disorders British journal of Occupational Therapy 2008 71 11 487 495

[B29] Eklund M Sandqvist G Psychometric properties of the Satisfaction with daily occupations (SDO) instrument and the Manchester short assessment of quality of life (MANSA) in women with scleroderma and without known illness Scandinavian Journal of Occupational Therapy 2006 13 23 30 10.1080/11038120500239578 16615412

[B30] Dolan P Modeling valuations for EuroQol health states Medical Care 1997 35 11 1095 1108 10.1097/00005650-199711000-00002 9366889

[B31] Dolan P Gudex C Kind P Williams A The time trade-off method: results from a general population study Health Economics 1996 5 141 154 10.1002/(SICI)1099-1050(199603)5:2<141::AID-HEC189>3.0.CO;2-N 8733106

[B32] Atroshi I Gummesson C Andersson B Dahlgren E Johansson A The disabilities of the arm, shoulder and hand (DASH) outcome questionnaire Acta Orthopaedica Scandinavica 2000 71 613 618 10.1080/000164700317362262 11145390

[B33] Hudak P Amadio P Bombardier C Development of an upper extremity outcome measure: the DASH (disabilities of the arm, shoulder and hand) American Journal of Industrial Medicine 1996 29 602 608 10.1002/(SICI)1097-0274(199606)29:6<602::AID-AJIM4>3.0.CO;2-L 8773720

[B34] Ware J Sherbourne C The MOS 36-item short-form health survey (SF-36) Medical Care 1992 30 6 473 483 10.1097/00005650-199206000-00002 1593914

[B35] Antonovsky A Hälsans mysterium 1991 Stockholm: Natur och Kultur

[B36] Antonovsky A The structure and properties of the sense of coherence scale Social Science Medicine 1993 36 6 725 733 10.1016/0277-9536(93)90033-Z 8480217

[B37] Kazis L Anderson J Meenan R Effect sizes for interpreting changes in health status Med Care 1989 27 178 189 10.1097/00005650-198903001-00015 2646488

[B38] Cohen J Statistical power analysis for the behaviour sciences 1977 New York: Academic Press

[B39] Portney L Watkins M Foundations of clinical research: applications to practise 2009 Third Upper Saddle River: Pearson Prentice Hall

[B40] Anke A Fugl-Meyer A Life satisfaction several years after severe multiple trauma - a retrospective investigation Clinical Rehabilitation 2003 17 431 442 10.1191/0269215503cr629oa 12785252

[B41] Eklund M Leufstadius C Relationships between occupational factors and health and well-being in individuals with persistant mental illness living in the community Canadian Journal of Occupational Therapy 2007 74 4 303 313 10.1177/000841740707400403 17985753

[B42] Håkansson C Svartvik L Lidfeldt J Nerbrand C Samsioe G Scherstén B Nilsson P Self-rated health in middle-aged women: Associations with sense of coherence and socioeconomic and health-related factors Scandinavian Journal of Occupational Therapy 2003 10 99 106 10.1080/11038120310009425 21275507

[B43] Eriksson M Lindström B Validity of Antonovsky's sense of coherence scale: a systematic review Journal of Epidemiology and Community Health 2005 59 460 466 10.1136/jech.2003.018085 15911640 PMC1757043

[B44] Cohen F al MJe Coping Behavioural health: a handbook of health enhancement and disease prevention 1984 New York: Wiley

[B45] Thomsen N Cederlund R Björk J Dahlin L Health-related quality of life in diabetic patients with carpal tunnel syndrome Diabetic Medicine 2010 27 466 472 10.1111/j.1464-5491.2010.02970.x 20536520

[B46] Holbrook T Anderson J Sieber W Browner D Hoyt D Outcome after major trauma: 12-months and 18-months follow-up results from the trauma recovery project The Journal of Trauma 1999 46 5 765 771 10.1097/00005373-199905000-00003 10338392

[B47] Strauser D Trauma symptomatology: implications for return to work Work 2008 31 2 245 252 18957742

[B48] Carlsson I Nilsson J Dahlin L Self-reported cold sensitivity in normal subjects and in patients with traumatic hand injuries or hand-arm vibration syndrome BMC Musculoskeletal Disorders 2010 in press 20462418 10.1186/1471-2474-11-89 PMC2881018

[B49] Ruijs A Jaquet J van Riel W Daanen H Hovius S Cold intolerance following median and ulnar nerve injuries: prognosis and predictors Journal of Hand Surgery (European volume) 2007 32 4 434 439 10.1016/j.jhsb.2007.02.012 17482322

[B50] Carlsson I Cederlund R Holmberg J Lundborg G Behavioural treatment of post-traumatic and vibration-induced digital cold sensitivity Scandinavian Journal of Plastic and Reconstructive and Hand Surgery 2003 37 6 371 378 10.1080/02844310310013055 15328778

[B51] Carlsson I Edberg A Wann-Hansson C Hand-injured patients' experiences of cold sensitivity and the consequences and adaptation for daily life: a qualitative study Journal of Hand Therapy 2009 23 1 53 61 10.1016/j.jht.2009.07.008 19765950

[B52] Gaston R Greenberg J Baltera R Mih A Hastings H Clinical outcomes of scaphoid and triquetral excision with capitolunate arthrodesis versus scaphoid excision and four-corner arthrodesis Journal of Hand Surgery (American Volume) 2009 34A 1407 1412 10.1016/j.jhsa.2009.05.018 19733983

[B53] Neidenbach P Audigé L Wilhelmi-Mock M Hansson B De Boer P The efficacy of closed reduction in displaced distal radius fractures Injury: International Journal of the Care of the Injured 2010 41 6 592 598 10.1016/j.injury.2009.10.055 19959165

[B54] Gummesson C Atroshi I Ekdahl C The disabilities of the arm, shoulder and hand (DASH) outcome questionnaire: longitudinal construct validity and measuring self-rated health change after surgery BMC Musculoskeletal Disorders 2003 4 11 10.1186/1471-2474-4-11 PMC165599 12809562

[B55] Townsend EPHJ Enabling occupation II: Advancing an occupational therapy vision for health, well-being, and justice through occupation 2007 Ottawa, Canada: CAOT Publications ACE

[B56] Guzelkucuk U Duman I Taskaynatan M Dincer K Comparison of therapeutic activities with therapeutic exercises in the rehabilitation of young adult patients with hand injuries Journal of Hand Surgery (American Volume) 2007 32A 1429 1435 10.1016/j.jhsa.2007.08.008 17996780

